# Higher TSH within the normal range is independently associated with reduced IVF clinical pregnancy odds: a K-means cluster analysis challenging the binary cutoff paradigm

**DOI:** 10.3389/fendo.2026.1856685

**Published:** 2026-10-23

**Authors:** Mohamed M. Amin, SI. Hassanein

**Affiliations:** 1 German University in Cairo, New Cairo City, Egypt; 2 Biochemistry Department, Faculty of Pharmacy and Biotechnology, German University in Cairo, New Cairo City, Egypt

**Keywords:** clinical pregnancy, euthyroid (normal TSH range), *in vitro* fertilization (IVF), K-means clustering, thyroid-stimulating hormone (TSH)

## Abstract

**Background:**

The impact of thyroid-stimulating hormone (TSH) variation within the normal reference range on IVF outcomes remains controversial, with prior studies yielding conflicting results.

**Methods:**

We conducted a retrospective cohort study of 878 euthyroid women (TSH 0.25–4.0 µIU/mL) undergoing fresh IVF/ICSI cycles at Al-Hayat Hospital, Iraq (2022–2024). Patients were classified into four groups using k-means clustering of serum TSH values: Cluster 1 (2.9–4.0 µIU/mL), Cluster 2 (2.2–2.8 µIU/mL), Cluster 3 (1.4–2.0 µIU/mL), and Cluster 4 (0.25–1.3 µIU/mL). Clinical, hormonal, and embryological parameters were compared across clusters, and stepwise logistic regression was performed to adjust for confounders.

**Results:**

Clinical pregnancy was achieved in 474/878 women (54%). As expected, women with pregnancy were younger (30.1 ± 5.1 vs. 31.2 ± 5.5 years, *p* = 0.003) and had lower mean TSH (1.95 ± 0.85 vs. 2.08 ± 0.96 µIU/mL, *p* = 0.030). Ovarian reserve and response markers were higher in the pregnancy group, including antral follicle count (AFC) (11.3 ± 4.2 vs. 10.4 ± 4.2, *p* = 0.001), preovulatory follicle count (PFC) (11.9 ± 4.2 vs. 11.0 ± 4.2, *p* = 0.003), and oocyte yield (10.4 ± 5.0 vs. 9.6 ± 4.8, *p* = 0.010). Oocyte maturation (80.5 ± 15.5% vs. 76.9 ± 17.1%, *p* = 0.003) and fertilization rates (88.0 ± 14.0% vs. 84.2 ± 16.9%, *p* = 0.003) were also significantly higher, while women without pregnancy required more ampoules (41.2 ± 18.8 vs. 36.6 ± 16.5, *p* < 0.001). Stepwise regression identified age (OR = 0.97, *p* = 0.059), ampoules (OR = 0.99, *p* = 0.024), oocyte yield (OR = 1.04, *p* = 0.014), maturation rate (OR = 1.10, *p* = 0.002), fertilization rate (OR = 1.06, *p* = 0.015), and TSH clusters as independent predictors. Cluster 3 (TSH 1.4–2.0 µIU/mL) showed the highest pregnancy rate (58.2%), while Cluster 1 (TSH 2.9–4.0 µIU/mL) had the lowest (42.7%) and was independently associated with reduced odds (OR = 0.53, 95% CI 0.35–0.80, *p* = 0.0024). Subgroup analysis revealed that unexplained and ovarian factor infertility were most sensitive to thyroid variation, whereas male factor infertility remained stable and PCO cases diverged.

**Conclusions:**

K-means clustering revealed that women with higher-normal TSH (Cluster 1: 2.9–4.0 µIU/mL) had independently reduced odds of clinical pregnancy (OR = 0.53, 95% CI 0.35–0.80, p = 0.002) compared with Cluster 3 (1.4–2.0 µIU/mL), challenging the adequacy of a single binary cutoff and supporting individualized, data-driven thyroid assessment in IVF.

## Introduction

Infertility affects an estimated 48.5 million couples worldwide (1), and assisted reproductive technology (ART) has become its primary treatment modality (2). *In vitro* fertilization and embryo transfer (IVF-ET) is a widely adopted treatment for infertility following mechanical or physiological abnormalities.

Thyroid-stimulating hormone (TSH) has been proposed to play a role in reproductive difficulties over several decades (3), including ovulatory dysfunction, infertility, miscarriage, and adverse maternal complications. Several studies have linked thyroid function to ovarian function and reproductive physiology. Thyroid hormones may influence folliculogenesis, estrogen and androgen metabolism, the menstrual cycle (4, 5), and endometrial receptivity (6). TSH is a peptide hormone produced by the anterior pituitary gland. It consists of two chains: an alpha and a beta chain. It has a molecular mass of approximately 28, 000 Da. Its composition is very similar to that of other glycoprotein hormones produced by the anterior pituitary gland. Luteinizing hormone (LH), follicle-stimulating hormone (FSH), and human chorionic gonadotropin (HCG) resemble TSH. Specifically, they all have the same alpha subunit as TSH but different beta chains that confer biological specificity to the hormone. As TSH, LH, FSH, and HCG share the same alpha subunit, they all function through the same cyclic adenine monophosphate (cAMP) second messenger system. The second cAMP messenger system involves the conversion of adenine monophosphate (AMP) to cAMP. In addition to cAMP, TSH activates the IP3 signaling cascade. The IP3 second messenger system involves calcium release from the sarcoplasmic reticulum (SR). Both cAMP and IP3/Ca2+ cascades lead to downstream physiological effects that enhance thyroid hormone synthesis and thyroid gland growth (7).

TSH serum concentrations above the reference values (0.25– 4 µIU/mL) have been associated with adverse reproductive and pregnancy outcomes, including infertility, miscarriages, prematurity, and congenital defects, even when FT4 is within the normal range (8). International guidelines on recurrent miscarriage uniformly recommend that TSH levels should be <2.5 µIU/mL in women with this diagnosis and hypothyroidism (9).

Subclinical hypothyroidism has been variably defined in the literature as TSH levels >2.5 μIU/mL, >4.5 μIU/mL, or above the upper limit of the assay-specific reference range (10, 11). Indeed, optimizing preconception upper reference TSH values for euthyroidism in a distinct population of subfertile or infertile women is under debate. In women undergoing ART, the prevalence of moderately elevated TSH levels (2.5–4.5 μIU/mL) was 20–26%, much higher than the prevalence of 5% in the general population (12, 13). Nevertheless, the effect of TSH variations within the reference values on reproductive outcomes remains unclear in patients without the aforementioned diagnoses (14, 15).

Meanwhile, there have been several reports on whether serum TSH levels above a specific cut-off result in unfavorable IVF outcomes. One study reported a significantly lower clinical PR in women with serum TSH >2.5 μIU/mL (16); however, other studies found no difference in the LBR when serum TSH 2.5 μIU/mL was used as a cutoff (17, 18). In a large retrospective cohort study including 1055 first IVF cycles, no differences were found in clinical PR, miscarriage rate, or delivery rate when serum TSH 2.5 or 4.5 μIU/mL was used as cut-off (19). One study also reported similar clinical PR, LBR, and miscarriage rates when a serum TSH level of 4.5 μIU/mL was used as the cut-off (20).

Many studies have focused on the relationship between pre-IVF serum TSH levels in infertile women and clinical IVF outcomes; however, their conclusions have been conflicting. In a meta-analysis by Zhao et al. (including 18 studies), clinical PR, LBR, and miscarriage rates were similar when TSH 2.5 μIU/mL was used as a cut-off (21). However, in that meta-analysis, when a TSH level of 3.5–5.0 μIU/mL was used as the cut-off, a significantly higher miscarriage rate (by 1.91 times) was found, but clinical PR and LBR were similar.

Despite this extensive literature, virtually all existing studies rely on arbitrary binary TSH cutoffs—most commonly 2.5 µIU/mL—which may obscure clinically meaningful gradations within the normal range. To our knowledge, no prior study has applied unsupervised machine-learning classification (k-means clustering) directly to pre-treatment TSH values to derive data-driven thyroid subgroups and evaluate their independent association with IVF pregnancy outcomes. The present study addresses this gap by using a cluster-based approach to stratify 878 euthyroid women undergoing IVF/ICSI, thereby challenging the binary cutoff paradigm with a continuous, patient-driven classification.

## Methods

### Study design

Data from patients who underwent fresh IVF/ICSI embryo transfers at Al-Hayat Hospital’s Infertility Unit, the Reproductive Medicine Center in Iraq (January 1, 2022, and December 31, 2024), were included in this retrospective cohort study.

### Patients and population

This study included women aged ≤40 years who underwent fresh IVF/ICSI cycles. Data on clinical, hormonal, and reproductive outcomes were obtained from the medical records. The inclusion criteria were as follows: FSH ≤12.0 IU/L, TSH between 0.25–4.0 μIU/mL, both ovaries present without abnormalities, transfer of Grade A embryos only, and no history of endocrine disease such as hyperprolactinemia. The exclusion criteria included autoimmune diseases, prior ovarian surgery, radio/chemotherapy, previous hormonal treatments (including LT4), uterine anomalies, sactosalpinx, untreated thyroid dysfunction (TSH <0.25 or >4 μIU/mL), history of diabetes mellitus (DM), hypertension (HTN), benign thyroid nodules (BTN), or chronic obstructive pulmonary disease (COPD), Obesity and poor embryo transfer.

A total of 878 patients met the inclusion criteria. Initially, the patients were divided into positive and negative pregnancy groups based on pregnancy outcomes. Finally, patients were categorized into four groups according to their serum TSH levels using a k-means clustering algorithm in R program, rather than arbitrary cut-off values. This mathematical classification yielded four distinct clusters: Cluster 1 (mean TSH ≈ 3.47 ± 0.33 μIU/mL), Cluster 2 (mean TSH ≈ 2.50 ± 0.22 μIU/mL), Cluster 3 (mean TSH ≈ 1.73 ± 0.23 μIU/mL), and Cluster 4 (mean TSH ≈ 0.92 ± 0.31 μIU/mL). Internal validation confirmed cluster stability: the silhouette coefficient was 0.549, the Calinski–Harabasz index was 3086.67, and 100% concordance was observed between K-means and hierarchical Ward-linkage solutions, indicating that the four-cluster partition is robust and not an artefact of algorithmic choice (see **Supplementary Table S1** for full validation metrics across k = 2–5).

Unfortunately, thyroid peroxidase antibody (TPA) and antithyroid antibody testing was not routinely performed in patients with TSH values between 0.25–4.0 μIU/mL with normal free T4, in line with local practice protocols.

### IVF/ICSI procedure

Patients underwent controlled ovarian stimulation using an individualized GnRH-agonist or GnRH-antagonist protocol. All patients underwent fresh (non-cryopreserved, same-stimulation-cycle) embryo transfer of Grade A embryos at either the cleavage stage (Day 3) or the blastocyst stage (Day 5), according to individual embryo development and clinician judgment.

Clinical pregnancy was defined as visualization of a gestational sac with a confirmed fetal heartbeat on transvaginal ultrasound performed at 6–7 weeks of gestation.

### Laboratory analysis

Serum TSH concentrations were determined using the VIDAS TSH assay (bioMérieux, Marcy-l’Étoile, France), which operates on the Enzyme-Linked Fluorescent Assay (ELFA) principle. The assay was performed using the VIDAS instrument, an automated immunoanalyzer platform designed for accurate and reproducible hormonal analysis. A reference range of 0.25 – 4.0 μIU/mL was adopted, in line with the manufacturer’s guidelines and relevant clinical literature. The assay demonstrated excellent reliability, with intra- and inter-assay coefficients of variation (CVs) maintained at ≤10%, thereby ensuring analytical precision across the measurement spectrum.

### Statistical analysis

The total sample size of 878 patients provided >90% statistical power (α = 0.05, two-tailed) to detect small effect sizes (Cohen’s *d* = 0.15), confirming that the cohort was sufficiently powered to evaluate whether normal-range TSH clusters, derived by k-means classification, were independently associated with clinical pregnancy outcomes. All analyses were performed using SPSS software, version 16.0 (SPSS Inc., Chicago, IL, USA), and cross-validated in R (version 4.5.0, R Foundation for Statistical Computing, Vienna, Austria). Descriptive statistics were calculated for all variables and are presented as means ± standard deviations for continuous variables and as frequencies with percentages for categorical variables. A one-way analysis of variance (ANOVA) was used to assess differences in IVF parameters across the four TSH clusters generated by k-means classification. This test compared the means of IVF-related outcomes (e.g., number of retrieved oocytes, fertilization rate, and embryo quality) among the mathematically defined TSH groups. For comparisons between binary outcome groups (patients with positive vs. negative clinical pregnancy), independent samples *t*-tests were conducted to evaluate differences in IVF parameters. Non-parametric tests (Mann–Whitney U) were applied for proportion-based variables such as oocyte maturation and fertilization rates. Binary logistic regression analysis was then performed to evaluate the association between TSH levels (both categorical clusters and continuous values) and clinical pregnancy outcomes. Odds ratios (ORs) with 95% confidence intervals (CIs) were calculated to quantify the strength of association between TSH and reproductive outcomes, while adjusting for potential confounders including age, antral follicle count, oocyte maturity, number of ampoules used for stimulation (1 ampoule = 75 IU), fertilization rate, and infertility etiology. A significance threshold of *p* < 0.05 was applied to all tests. Assumptions of normality and homogeneity of variances were assessed prior to conducting parametric analyses. Justification for clustering approach: K-means classification was selected to delineate TSH subgroups because it provides a data-driven method of identifying natural partitions within the continuous distribution of TSH values. Unlike arbitrary dichotomization (e.g., <2.5 vs. ≥2.5 µIU/mL), clustering captures subtle gradations across the normal range and ensures balanced group sizes, thereby improving statistical power and reducing bias misclassification. This approach allows for a more nuanced evaluation of how thyroid status interacts with ovarian response and pregnancy outcomes. Ovarian stimulation protocol variables were additionally compared across TSH clusters; no significant differences were observed (all p > 0.05), confirming that stimulation intensity did not confound the TSH–pregnancy association (see **Supplementary Table S2**).

## Results

Of the 878 patients included, 474 (54%) achieved clinical pregnancy, while 404 (46%) did not. As shown in **Table 1** and illustrated in **Figure 1**, women with positive outcomes were significantly younger (30.1 ± 5.1 vs. 31.2 ± 5.5 years, *p* = 0.003) and had lower mean TSH levels (1.95 ± 0.85 vs. 2.08 ± 0.96 μIU/mL, *p* = 0.030). Ovarian reserve and response markers were consistently higher in the pregnancy group, including antral follicle count (*p* = 0.001), preovulatory follicle count (*p* = 0.003), number of oocytes collected (*p* = 0.010), and oocyte maturation rate (80.5 ± 15.5% vs. 76.9 ± 17.1%, *p* = 0.003, Mann–Whitney). Fertilization rate was also higher (88.0 ± 14.0% vs. 84.2 ± 16.9%), reaching statistical significance (*p* = 0.003, Mann–Whitney). In contrast, women who did not achieve pregnancy required significantly more ampoules for stimulation (41.2 ± 18.8 vs. 36.6 ± 16.5, *p* < 0.001). Other parameters, including BMI, gonadotropins, endometrial thickness, and hormone levels, showed no significant differences. Effect size analysis confirmed that oocyte maturation, fertilization outcomes, and ovarian reserve markers had the strongest associations with pregnancy.

**Table 1:** Comparison of IVF parameters between positive and negative clinical pregnancy groups among women with normal TSH levels (0.25–4.0 µIU/mL).

Variable	Mean _Pos	Mean _Neg	Test	p-value	Sig	Cohens_d
age	30.13 ± 5.11	31.20 ± 5.52	t-test	0.003	**	-0.20
BMI	25.02 ± 3.07	25.11 ± 2.87	t-test	0.655		-0.03
FSH	6.06 ± 2.56	6.05 ± 2.57	t-test	0.937		0.01
E21	37.41 ± 15.30	36.34 ± 15.97	t-test	0.312		0.07
LH	3.84 ± 2.16	3.65 ± 2.14	t-test	0.201		0.09
prolactin	19.69 ± 8.21	19.67 ± 8.38	t-test	0.971		0.00
TSH	1.95 ± 0.85	2.08 ± 0.96	t-test	0.030	*	-0.15
AFC	11.31 ± 4.20	10.39 ± 4.21	t-test	0.001	**	0.22
PFC	11.88 ± 4.16	11.02 ± 4.17	t-test	0.003	**	0.20
ampoules	36.61 ± 16.45	41.22 ± 18.82	t-test	<0.001	***	-0.26
days	10.74 ± 1.61	10.95 ± 1.74	t-test	0.075		-0.12
endometrium	9.35 ± 1.56	9.37 ± 1.58	t-test	0.831		-0.01
pro2	0.91 ± 0.33	0.91 ± 0.33	t-test	0.959		0.00
E2	2391.84 ± 917	2291.69 ± 1058	t-test	0.138		0.10
EC	10.41 ± 4.97	9.55 ± 4.81	t-test	0.010	**	0.17
Maturation rate	80.54 ± 15.54%	76.91 ± 17.10%	Mann–Whitney	0.003	**	0.22
Fertilization rate	88.02 ± 13.96%	84.24 ± 16.87%	Mann–Whitney	0.003	**	0.25

Data are presented as mean ± standard deviation (SD). P-values were calculated using independent samples t-tests to compare the means between the groups. Significance levels: p < 0.05 (*), p < 0.01 (**), p < 0.001 (***). TSH, Thyroid-Stimulating Hormone; FSH, Follicle-Stimulating Hormone; LH, Luteinizing Hormone; E2, Estradiol; EC, Egg collected; AFC= antral follicle count; PFC, preovulatory follicle count.

**Figure 1:**
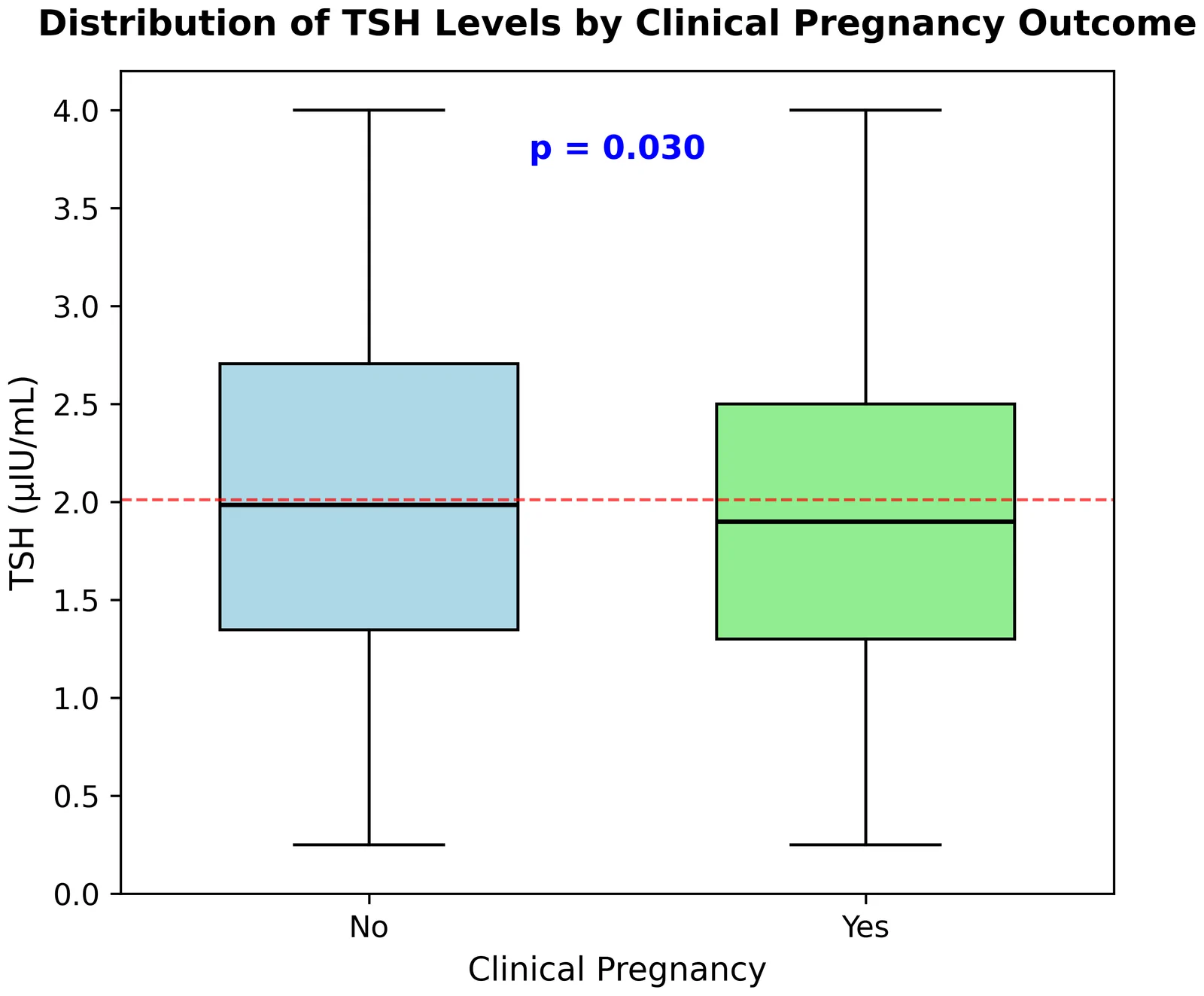
Distribution of thyroid-stimulating hormone (TSH) levels in patients with and without clinical pregnancy. Box plot comparing serum TSH concentrations (µIU/mL) between women who achieved clinical pregnancy (n = 474) and those who did not (n = 404) among 878 euthyroid women (TSH 0.25–4.0 µIU/mL) undergoing fresh IVF/ICSI cycles. Women with clinical pregnancy had significantly lower median TSH and a more compact interquartile range compared with non-pregnant women (mean 1.95 ± 0.85 vs. 2.08 ± 0.96 µIU/mL; independent samples t-test, p = 0.030). The central line represents the median, box edges indicate the 25th and 75th percentiles, and whiskers extend to 1.5× the interquartile range.

**Figure 1** illustrates these findings by showing the distribution of thyroid-stimulating hormone (TSH) levels in women who achieved clinical pregnancy compared with those who did not. Notably, the median TSH value was slightly lower among those with successful outcomes, and the interquartile range (IQR) was more compact, reflecting less variability in TSH levels in the pregnancy group. This subtle tendency toward lower and more consistent TSH values among pregnant patients underscores the possible influence of optimal thyroid function on the success of IVF.

Box plot displays the distribution of thyroid-stimulating hormone (TSH) levels among patients who achieved clinical pregnancy (“Yes”) and those who did not (“No”).

Because ovarian reserve and response markers are biologically interrelated and known to influence IVF success, we performed multivariate logistic regression adjusting for these established confounders. As shown in **Table 2** and illustrated in **Figure 2**, the model confirmed that well-established predictors performed as expected: younger age (OR = 0.97, 95% CI 0.95–1.00, p = 0.046), higher oocyte yield (OR = 1.04, 95% CI 1.01–1.08, p = 0.010), greater maturation rate (OR = 1.10, 95% CI 1.03–1.16, p = 0.002), and higher fertilization rate (OR = 1.06, 95% CI 1.01–1.12, p = 0.015) all favored pregnancy, while greater stimulation intensity (ampoules; OR = 0.99, 95% CI 0.98–1.00, p = 0.034) was inversely associated—reflecting that patients requiring more gonadotropin stimulation tend to have poorer ovarian response. Critically, after adjusting for all these recognized factors, TSH remained an independent predictor of clinical pregnancy (OR = 0.84, 95% CI 0.72–0.98, p = 0.025), indicating that lower-normal TSH values confer a significant advantage beyond what patient age and ovarian efficiency alone explain.

**Table 2:** Multivariate logistic regression predicting clinical pregnancy.

Variable	OR	Lower	Upper	P- value
age	0.97	0.95	1.00	0.0464 *
TSH	0.84	0.72	0.98	0.0253 *
ampoules	0.99	0.98	1.00	0.0342 *
EC	1.04	1.01	1.08	0.0095 **
Maturation rate logit	1.10	1.03	1.16	0.0023 **
Fertilization rate logit	1.06	1.01	1.12	0.0153 *

Significance codes: ***p < 0.001, **p < 0.01, *p < 0.05,. p < 0.1.

**Figure 2:**
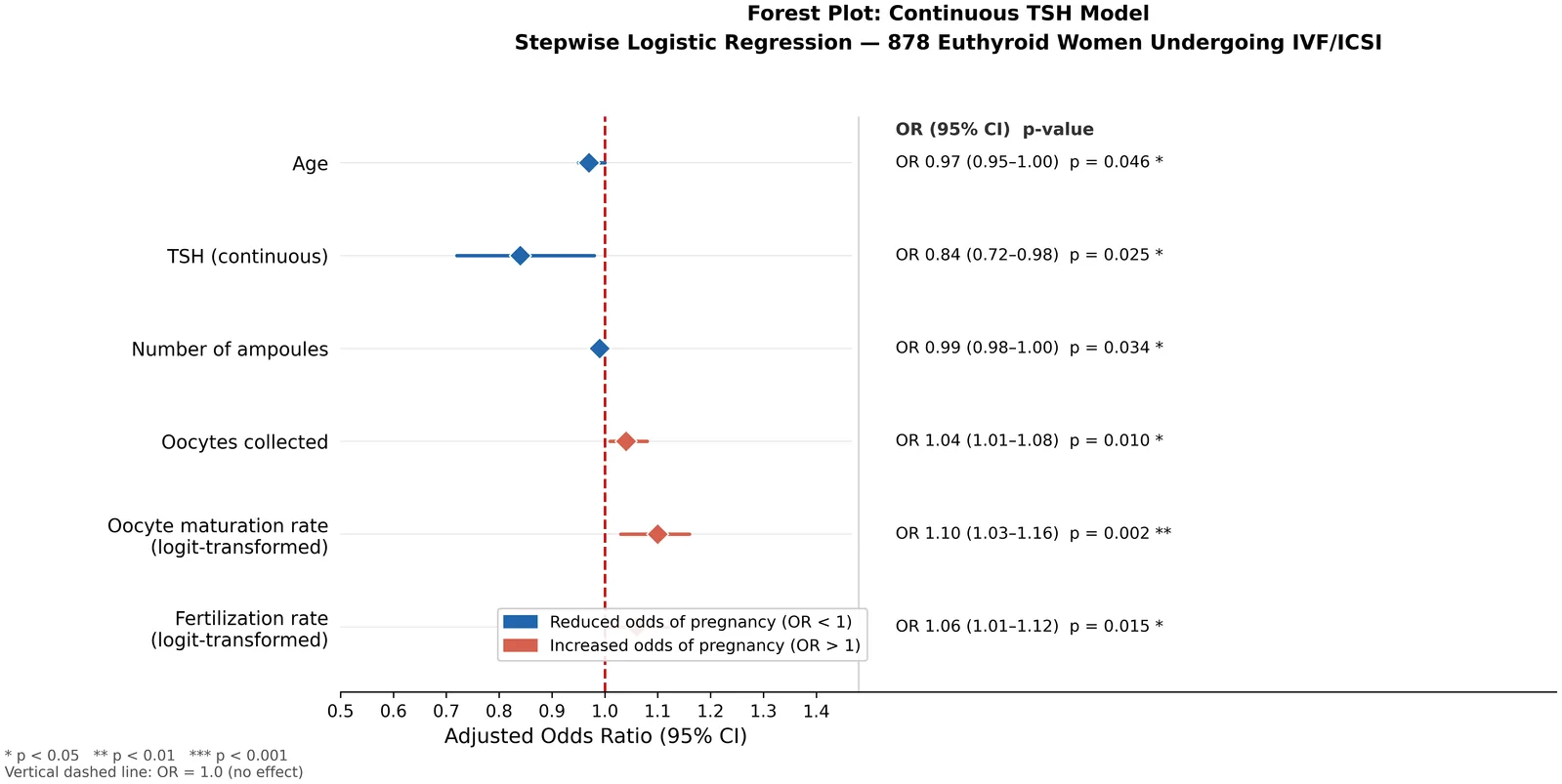
Forest plot of adjusted odds ratios from multivariate logistic regression predicting clinical pregnancy (continuous TSH model). Adjusted odds ratios (ORs) with 95% confidence intervals for independent predictors of clinical pregnancy identified by stepwise binary logistic regression in 878 euthyroid women undergoing IVF/ICSI. Predictors include: age (OR = 0.97, 95% CI 0.95–1.00, p = 0.046), TSH as a continuous variable (OR = 0.84, 95% CI 0.72–0.98, p = 0.025), number of ampoules (OR = 0.99, 95% CI 0.98–1.00, p = 0.034), oocytes collected (OR = 1.04, 95% CI 1.01–1.08, p = 0.010), oocyte maturation rate (logit-transformed; OR = 1.10, 95% CI 1.03–1.16, p = 0.002), and fertilization rate (logit-transformed; OR = 1.06, 95% CI 1.01–1.12, p = 0.015). The vertical dashed line indicates OR = 1.0 (no effect); markers to the left indicate reduced odds and to the right indicate increased odds of clinical pregnancy.

K-means clustering of serum TSH values delineated four distinct groups with mean concentrations of Cluster 1: 3.47 µIU/mL (range 2.9–4.0), Cluster 2: 2.50 µIU/mL (range 2.2–2.8), Cluster 3: 1.73 µIU/mL (range 1.4–2.0), and Cluster 4: 0.92 µIU/mL (range 0.25–1.3). ANOVA confirmed highly significant differences in TSH distribution across clusters (F = 3041, *p* < 0.001), thereby validating the robustness of the classification. Importantly, patient distribution was balanced, with 146 in Cluster 1, 225 in Cluster 2, 283 in Cluster 3, and 224 in Cluster 4, ensuring adequate statistical power for comparisons.

As presented in **Table  3**, the comparison of baseline and treatment characteristics across the four clusters revealed that age, BMI, gonadotropin levels (FSH, LH, and E2 on day 2), and endometrial thickness did not differ significantly. In contrast, serum TSH values were clearly stratified by cluster (*p* < 0.001), underscoring the discriminatory power of the grouping. Ovarian reserve and response indicators, including AFC, PFC, and stimulation characteristics (ampoules and days), showed no meaningful variation. Subtle differences were observed in endocrine and embryology outcomes: estradiol at trigger tended to be lower in Cluster 2 compared with Clusters 1 and 3 (*p* = 0.074), while the number of oocytes retrieved and maturation rates exhibited modest but non-significant variation. Fertilization rates remained comparable across clusters (*p* = 0.886). Collectively, these findings highlight that thyroid function was the principal determinant of cluster separation, with only limited downstream effects on ovarian and embryological parameters.

**Table 3:** Comparison of IVF parameters across TSH level groups in women undergoing assisted reproduction (TSH 0.25–4.0 µIU/mL).

Variable	Cluster_1	Cluster_2	Cluster_3	Cluster_4	P_value
Number	146	225	283	224	
age	30.60 ± 5.39	30.47 ± 5.34	30.47 ± 5.46	30.97 ± 5.12	0.712
BMI	25.08 ± 3.14	25.28 ± 2.87	24.95 ± 2.98	24.95 ± 3.00	0.584
FSH	6.27 ± 2.43	6.09 ± 2.59	5.97 ± 2.47	6.00 ± 2.73	0.665
E21	37.63 ± 17.49	37.03 ± 16.02	37.49 ± 14.96	35.60 ± 14.71	0.514
LH	3.92 ± 2.27	3.85 ± 2.17	3.66 ± 2.10	3.66 ± 2.10	0.502
TSH	3.47 ± 0.33	2.50 ± 0.22	1.73 ± 0.23	0.92 ± 0.31	<0.001 ***
AFC	10.71 ± 4.64	10.81 ± 4.36	11.19 ± 4.16	10.70 ± 3.90	0.520
PFC	11.16 ± 4.41	11.36 ± 3.97	11.55 ± 4.14	11.73 ± 4.33	0.596
ampoules	38.17 ± 18.75	39.78 ± 19.19	38.87 ± 17.61	37.85 ± 15.54	0.683
days	10.74 ± 1.75	10.89 ± 1.66	10.89 ± 1.60	10.78 ± 1.74	0.734
endometrium	9.26 ± 1.61	9.42 ± 1.58	9.37 ± 1.71	9.35 ± 1.34	0.795
pro2	0.90 ± 0.34	0.93 ± 0.33	0.93 ± 0.34	0.87 ± 0.31	0.220
E2	2156.61 ± 844	2412.14 ± 1087	2387.81 ± 866	2349.23 ± 1090	0.074
EC	9.55 ± 4.95	10.13 ± 5.37	10.48 ± 5.07	9.61 ± 4.13	0.142
Maturation rate	78.99 ± 16.65%	79.45 ± 16.42%	77.42 ± 16.35%	80.04 ± 16.12%	0.214
Fertilization rate	86.84 ± 15.05%	86.40 ± 15.84%	85.93 ± 15.25%	86.25 ± 15.75%	0.886

Comparison of parameters across TSH clusters (mean ± SD). P-values are from global one-way ANOVA (continuous variables) or chi-square test (categorical variables) comparing all four clusters simultaneously. Significance levels: *p < 0.05, **p < 0.01, ***p < 0.001. For variables marked ***, all six *post-hoc* pairwise comparisons (Bonferroni-corrected) also reached p < 0.001.

Data are presented as mean ± standard deviation. Differences across TSH clusters were assessed using one-way analysis of variance (ANOVA or Kruskal-Wallis). Significance levels are indicated as follows: p < 0.05, p < 0.01, p < 0.001 (***). Cluster definitions: Cluster I, mean TSH 3.47 µIU/mL (range 2.9–4.0); Cluster II, mean TSH 2.50 µIU/mL (range 2.2–2.8); Cluster III, mean TSH 1.73 µIU/mL (range 1.4–2.0); Cluster IV, mean TSH 0.92 µIU/mL (range 0.25–1.3). TSH, Thyroid-Stimulating Hormone; FSH, Follicle-Stimulating Hormone; LH, Luteinizing Hormone; E2= Estradiol.

The **Figure 3** and **Table 4** together demonstrate that pregnancy outcomes varied significantly across the four TSH clusters. The bar chart shows a global Chi-square p = 0.0268, with a notable difference between Cluster 1 and Cluster 3 (p = 0.024), indicating that clinical pregnancy rates were not evenly distributed across groups. **Table 4** complements this by detailing pregnancy rates within specific infertility categories: for example, male factor infertility showed relatively high rates across all clusters (≈52–63%), while age-related infertility was lowest in Cluster 2 (12.5%) but higher in Clusters 3 and 4 (≈46–42%). Polycystic ovary cases had the highest pregnancy rate in Cluster 1 (66.7%), whereas unexplained infertility showed increasing rates from Cluster 1 (34.3%) to Cluster 3 (65.6%). The overall pregnancy rate rose from 42.7% in Cluster 1 to 58.2% in Cluster 3, before slightly declining in Cluster 4 (56.0%). Taken together, the figure highlights the statistical significance of differences across clusters, while the table provides granular detail by infertility factor, reinforcing that thyroid function stratification was associated with meaningful variation in clinical pregnancy outcomes.

**Figure 3:**
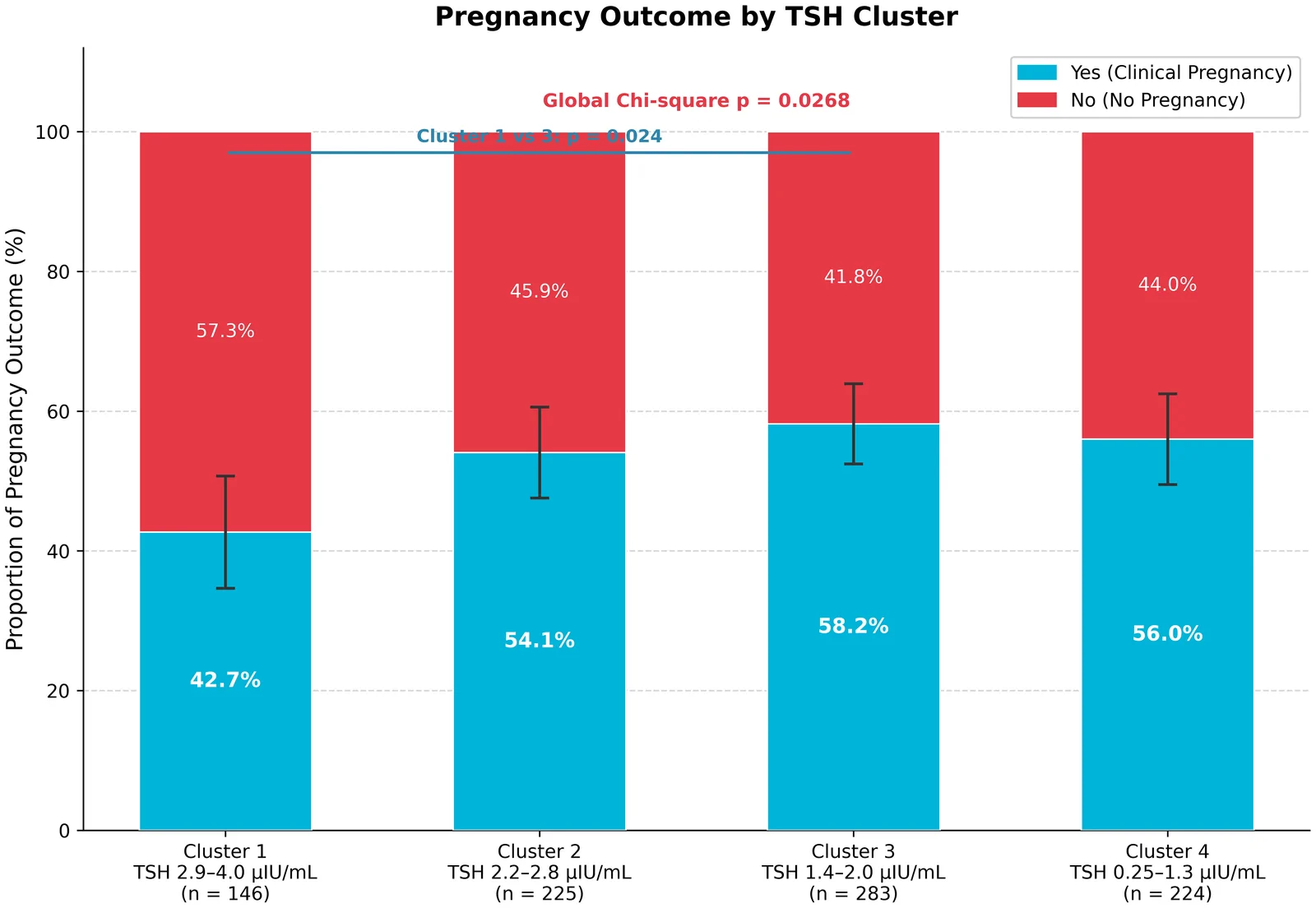
Clinical pregnancy rates across four TSH clusters defined by k-means classification. Bar chart showing clinical pregnancy rates (%) for each of four TSH clusters generated by k-means clustering of pre-treatment serum TSH values in 878 euthyroid women undergoing IVF/ICSI: Cluster 1 (TSH 2.9–4.0 µIU/mL, mean 3.47, n = 146, pregnancy rate = 42.7%), Cluster 2 (TSH 2.2–2.8 µIU/mL, mean 2.50, n = 225, pregnancy rate = 54.1%), Cluster 3 (TSH 1.4–2.0 µIU/mL, mean 1.73, n = 283, pregnancy rate = 58.2%), and Cluster 4 (TSH 0.25–1.3 µIU/mL, mean 0.92, n = 224, pregnancy rate = 56.0%). Overall differences were significant (Chi-square test, p = 0.027), with a notable pairwise difference between Cluster 1 and Cluster 3 (p = 0.024). Error bars represent 95% confidence intervals for proportions.

**Table 4:** Pregnancy rates by infertility factor across TSH level groups (TSH 0.25–4.0 µIU/mL).

Factor	Cluster 1	Cluster 2	Cluster 3	Cluster 4
age	30.0%	12.5%	46.1%	41.6%
combine	40.0%	46.6%	50.0%	50.0%
male	52.1%	51.3%	55.3%	62.5%
Normal (gender selection)	37.5%	64.3%	60.0%	57.5%
ovary	38.9%	56.0%	63.00%	40.0%
pco	66.6%	58.3%	57.9%	42.8%
unexplained	34.3%	59.2%	65.6%	61.3%
Total	42.65%	54.1%	58.2%	56.0%

Pregnancy rates per infertility factor across TSH clusters.

Values represent the percentage of patients who achieved clinical pregnancy within each infertility category and TSH cluster. Pregnancy rate (%) was calculated as (Number of Clinical Pregnancies ÷ Total Cases in Group) × 100. TSH = Thyroid-Stimulating Hormone; PCO = Polycystic Ovary, Normal= gender selection. K-means clustering of serum TSH values delineated four distinct groups with mean concentrations of cluster1 3.47 µIU/mL (range 2.9–4.0), cluster2 2.50 µIU/mL (range 2.2–2.8), cluster3 1.73 µIU/mL (range 1.4–2.0), and cluster4 0.92 µIU/mL (range 0.25–1.3), respectively.

To further validate these observations, we performed multivariate logistic regression analyses using categorical TSH clusters, adjusting for age, ovarian reserve markers, and stimulation parameters. This approach allowed us to determine whether TSH remained an independent predictor of clinical pregnancy after controlling for confounding factors and to quantify the relative risk associated with each stratum.

As shown in **Table 5** and illustrated in **Figure 4**, the stepwise logistic regression analysis revealed that several clinical parameters within the normal TSH range were independently associated with pregnancy outcomes. Age exerted a modest but significant negative effect (OR = 0.97, *p* = 0.036), while higher gonadotropin consumption (ampoules, OR = 0.99, *p* = 0.030) was also linked to lower odds, reflecting reduced ovarian responsiveness in patients requiring more stimulation. Egg collection (EC, OR = 1.04, *p* = 0.015) showed a positive association, indicating that retrieving more oocytes increased the likelihood of pregnancy. Stronger predictors were observed for oocyte maturation rate (logit-transformed; OR = 1.10, *p* = 0.002) and fertilization rate (logit-transformed; OR = 1.07, *p* = 0.014), both of which significantly improved pregnancy outcomes, underscoring the importance of gamete and embryo competence.

**Table 5:** Stepwise logistic regression results.

Predictor	Odds ratio	Lower 95% CI	Upper 95% CI	p-value
age	0.97	0.95	1.00	0.0588
ampoules	0.99	0.98	1.00	0.0238 *
EC	1.04	1.01	1.07	0.0144 *
Maturation rate logit	1.10	1.03	1.16	0.00203 **
Fertilization rate logit	1.07	1.01	1.12	0.0143 *
TSH group kmeansCluster 1	0.53	0.35	0.80	0.0024 **
TSH group kmeansCluster 2	0.84	0.59	1.21	0.353
TSH group kmeansCluster 4	0.88	0.61	1.26	0.474

Data are presented as odds ratios (OR) with 95% confidence intervals and p-values. Significance levels: *p<0.05, **p<0.01, ***p<0.001.

**Figure 4:**
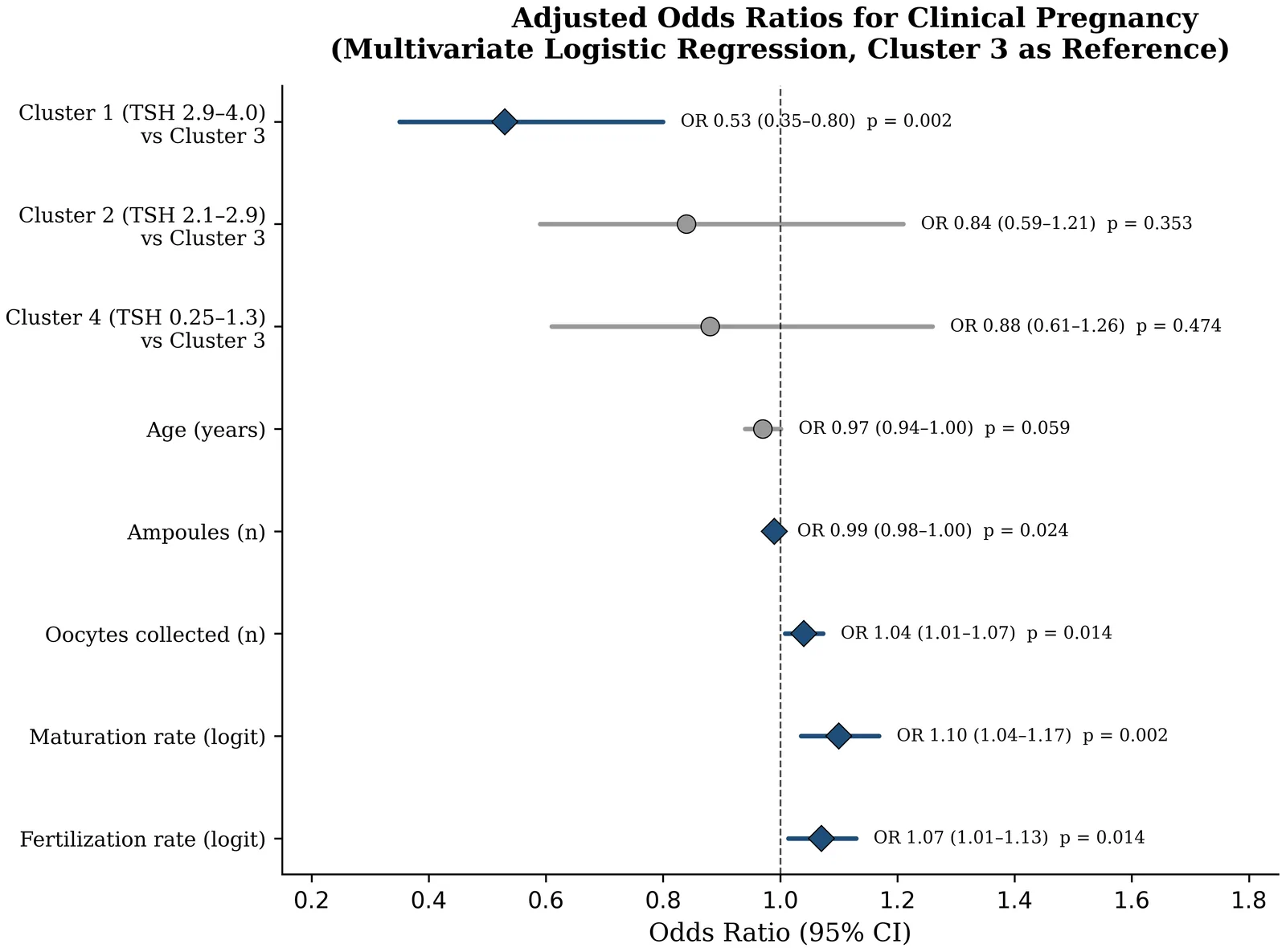
Forest plot of adjusted odds ratios from stepwise logistic regression predicting clinical pregnancy (categorical TSH cluster model). Adjusted odds ratios (ORs) with 95% confidence intervals for independent predictors of clinical pregnancy using TSH clusters as categorical variables, with Cluster 3 (TSH 1.4–2.0 µIU/mL, n = 283) as the reference group. Covariates adjusted: age (OR = 0.97, p = 0.059), ampoules (OR = 0.99, p = 0.024), oocytes collected (OR = 1.04, p = 0.014), oocyte maturation rate (logit-transformed; OR = 1.10, p = 0.002), and fertilization rate (logit-transformed; OR = 1.07, p = 0.014). Cluster-specific ORs relative to Cluster 3: Cluster 1 (OR = 0.53, 95% CI 0.35–0.80, p = 0.002), Cluster 2 (OR = 0.84, 95% CI 0.59–1.21, p = 0.353), Cluster 4 (OR = 0.88, 95% CI 0.61–1.26, p = 0.474). Only Cluster 1 showed a statistically significant independent reduction in pregnancy odds. The vertical dashed line indicates OR = 1.0 (no effect). Significance levels: *p < 0.05, **p < 0.01.

Regarding TSH clustering, patients in Cluster 1 (mean TSH ≈ 3.5 µIU/mL) had significantly lower pregnancy odds compared with the reference Cluster 3 (OR = 0.53, *p* = 0.002), while Clusters 2 and 4 did not differ significantly. Overall, these findings emphasize that reproductive efficiency markers (egg collection, maturation, and fertilization rates) are the strongest predictors of success, and that variation within the normal TSH range may still influence pregnancy outcomes, with higher values in Cluster 1 associated with reduced odds.

## Discussion

Our findings suggest that subtle variation in TSH within the normal reference range may be associated with IVF outcomes, particularly when considered alongside ovarian response markers. Stepwise logistic regression confirmed that several parameters were independently associated with pregnancy, including age, gonadotropin ampoules, oocyte yield, maturation rate, fertilization rate, and TSH. This indicates that lower-normal TSH values remain relevant even after accounting for other reproductive efficiency markers, underscoring their potential role in reproductive endocrinology. These results are consistent with recent reports suggesting that thyroid status, even within reference limits, can modulate ovarian responsiveness and IVF success (22–25).

The stratified analysis further supports this association. Pregnancy rates differed significantly across TSH clusters generated by k-means classification, with Cluster 3 (TSH 1.4–2.0 µIU/mL) showing the most favorable outcomes and Cluster 1 (TSH 2.9–4.0 µIU/mL) the least favorable. Importantly, regression analysis confirmed that Cluster 1 was independently associated with reduced odds of pregnancy (OR = 0.53, 95% CI 0.35–0.80, p = 0.0024), while Clusters 2 and 4 did not differ significantly from Cluster 3. These associations reinforce that thyroid status, even within reference limits, is linked to variation in reproductive outcomes, and that higher-normal TSH values may correspond to less favorable IVF success.

Subgroup analysis revealed that the impact of TSH variation differs across infertility etiologies. The most favorable outcomes were observed in Cluster 3, particularly in unexplained and ovarian factor infertility. In contrast, Cluster 1 was associated with lower pregnancy rates across most categories, suggesting that higher-normal TSH values may correspond to less favorable reproductive outcomes. Male factor infertility showed relatively stable pregnancy rates across clusters, while PCO cases diverged from the general pattern, with unexpectedly high success noted in Cluster 1. This variation may reflect the complex endocrine environment in women with polycystic ovary syndrome, where compensatory mechanisms or stimulation protocols could mitigate the impact of higher-normal TSH. These subgroup associations suggest that thyroid status interacts with specific infertility etiologies.

Comparison with the literature highlights both concordance and divergence. Zhou et al. (26) and Fazeli et al. (3) reported improved outcomes in women with TSH < 2.5 µIU/mL, particularly in IVF/ICSI settings, supporting the concept that lower‐normal thyroid function may be advantageous. In contrast, Negreiros d’Assunção et al. (27), in a large retrospective cohort and meta‐analysis including over 13, 000 women, found no significant differences in IVF outcomes between women with TSH 0.5–2.49 mIU/L and those with 2.5–4.5 mIU/L, concluding that variation within the normal range does not impact IVF success in euthyroid women without thyroid autoimmunity. Similarly, Nádasdi et al. (28), using random-forest machine learning on 1, 382 IVF cycles, found that pre-treatment TSH contributed negligible predictive importance for clinical pregnancy, further challenging the clinical utility of TSH as a standalone predictor. While the scale of these analyses is impressive, several methodological aspects may have obscured clinically relevant associations: broad dichotomization (<2.5 vs. 2.5–4.5 mIU/L) fails to capture subtle gradations, pooling heterogeneous populations dilutes subgroup‐specific effects, and lack of adjustment for ovarian response markers limits detection of independent contributions. By contrast, our cluster‐based and adjusted analysis demonstrated that higher‐normal TSH (≥3.0–4.0 µIU/mL, Cluster 1) was independently associated with diminished pregnancy odds, while Cluster 3 consistently showed the most favorable outcomes. This suggests that methodological refinement—through clustering and multivariate adjustment—reveals clinically significant patterns that pooled analyses overlook (29). Other studies further illustrate the complexity of this relationship: Venables et al. (30) found no association in IUI populations, but their focus on autoimmunity and low baseline success rates (~10%) may have masked thyroid effects; Coussa et al. (31) likewise reported no justification for enforcing a cutoff of ≤2.5 µIU/mL, but their design was restricted to IVF‐conceived pregnancies and relied on broad categories, limiting generalizability. Repelaer van Driel-Delprat et al. (32) similarly found no association between TSH 0.4–2.5 vs. 2.5–4.0 mIU/L and live birth in 4, 894 IVF/ICSI cycles, although they noted a possible subfertility-diagnosis interaction. The largest interventional evidence—the TABLET trial (33, N = 952 euthyroid women, NEJM)—demonstrated that levothyroxine supplementation in women with thyroid autoimmunity did not improve live birth rates, casting doubt on whether TSH optimization alone, without addressing autoimmune mechanisms, can improve outcomes (34).

Taken together, these findings highlight that while the underlying infertility diagnosis remains a primary determinant of IVF success, thyroid status within the normal range is also associated with pregnancy outcomes across most diagnostic categories. The divergence observed in PCO cases, where higher-normal TSH was linked to unexpectedly favorable outcomes, underscores the complexity of endocrine interactions and the need for subgroup-specific evaluation. Overall, our results are consistent with the trend reported in recent guideline recommendations (35, 36), which advocate tighter TSH control in women undergoing fertility treatment. However, rather than supporting a universal cutoff of < 2.5 µIU/mL, our findings emphasize that individualized thyroid assessment—guided by data-driven stratification, infertility context, and ovarian response parameters—may be most appropriate in reproductive medicine.

### Limitations

Several limitations must be acknowledged. First, the retrospective design precludes causal inference and may be subject to unmeasured confounding. Although we adjusted for age, ovarian reserve, stimulation parameters and BMI, other factors such as iodine status and subtle endocrine interactions were not captured. Second, our primary endpoint was clinical pregnancy rather than live birth. While clinical pregnancy is a reliable and routinely documented outcome, live birth remains the definitive measure of IVF success, and future longitudinal studies are needed. Third, thyroid autoantibodies (anti-TPO, anti-Tg) were not consistently available in our dataset. Given their potential independent impact on implantation and miscarriage risk, omission of these markers limits the scope of thyroid assessment. Finally, the study population was restricted to women with TSH values within the normal range, and results may not be generalizable to overt thyroid dysfunction.

### Clinical implications and future directions

Despite these limitations, our findings have important clinical implications. They indicate that maintaining TSH within the lower to mid-normal range (approximately 1.0–2.99 µIU/mL) is *associated* with more favorable IVF outcomes, whereas levels ≥3.0–4.0 µIU/mL are *associated* with diminished success. These results reinforce the importance of thyroid monitoring in women undergoing assisted reproduction and support consideration of thyroid optimization strategies prior to treatment. Importantly, our data demonstrates an association rather than causation, and prospective studies incorporating comprehensive thyroid panels, iodine status, and long-term live birth outcomes are warranted to validate these observations and refine clinical guidelines. Furthermore, mechanistic studies exploring the interplay between thyroid hormones, prolactin, and ovarian physiology may provide deeper insights into the endocrine pathways that may contribute to reproductive success.

## Conclusion

This retrospective cohort study of 878 euthyroid women undergoing IVF shows that variation in serum TSH within the normal reference range is associated with differences in reproductive outcomes. K-means clustering identified four distinct groups, with Cluster 3 (TSH 1.4–2.0 µIU/mL) linked to the most favorable pregnancy rates and Cluster 1 (2.9–4.0 µIU/mL) associated with reduced success, even after adjustment for age, ovarian reserve, and stimulation parameters. Subgroup analysis further indicated that unexplained and ovarian factor infertility were most sensitive to thyroid variation, while male factor infertility remained stable and PCO cases diverged.

These findings highlight thyroid status as a relevant cofactor in assisted reproduction and support individualized thyroid assessment rather than reliance on universal cutoffs. While consistent with the trend in recent guidelines advocating tighter TSH control (35–37), our results emphasize association only, not causation, and suggest that maintaining TSH in the lower-normal range may be advantageous in select infertility subgroups. Prospective studies incorporating comprehensive thyroid panels, iodine status, and live birth outcomes are needed to confirm these associations and clarify underlying mechanisms.

## Data Availability

The original contributions presented in the study are included in the article/**Supplementary Material**. Further inquiries can be directed to the corresponding author.
