# Supplementary material for: Higher TSH within the normal range is independently associated with reduced IVF clinical pregnancy odds: a K-means cluster analysis challenging the binary cutoff paradigm
Source: Front Endocrinol (Lausanne). 2026 Oct 23;17:1856685. doi: 10.3389/fendo.2026.1856685 (PMC13739597; doi:10.3389/fendo.2026.1856685)
Supplement: Supplementary file 1 [file DataSheet1.pdf]

Supplementary Material

Higher TSH Within the Normal Range Is Independently Associated with Reduced IVF Clinical Pregnancy Odds: A K-Means Cluster Analysis Challenging the Binary Cutoff Paradigm

Supplementary Methods

K-means clustering was applied to thyroid-stimulating hormone (TSH) values (mIU/L) using the 1-dimensional feature space. The optimal number of clusters (k) was determined through evaluation of multiple internal validity indices across k = 2 to 5, combined with clinical interpretability of the resulting TSH intervals.

Three complementary internal validity indices were calculated: (1) Silhouette coefficient — measures cohesion and separation (higher is better, maximum 1.0); (2) Calinski-Harabasz (CH) index — ratio of between-cluster to within-cluster dispersion (higher indicates better-defined clusters); and (3) Dunn index — ratio of minimum inter-cluster distance to maximum intra-cluster diameter (higher is better). External validation was performed by comparing k-means assignments against agglomerative hierarchical clustering (Ward linkage) and against natural TSH quartile groups.

To verify that stimulation protocol did not confound the association between TSH clusters and clinical pregnancy, gonadotropin type, total ampoules used (1 ampoule = 75 IU), stimulation duration (days), and peak estradiol (E2, pg/mL) were compared across the four TSH clusters using the Kruskal-Wallis test for continuous variables and Chi-square test for categorical variables.

Supplementary Table S1. K-Means Clustering Validation Metrics

Comparison of internal validity indices across k = 2 to 5 clusters. k = 4 was selected based on the highest Calinski-Harabasz index and optimal clinical interpretability of the resulting TSH intervals (0–1, 1–2, 2–3, 3–4 mIU/L). External validation showed 100% concordance with hierarchical clustering and 85.1% concordance with natural TSH quartiles.

k	Silhouette Coefficient	Calinski-Harabasz Index	Dunn Index	WCSS	Selection
2	0.584	1,883.67	0.0053	227.40	—
3	0.563	2,484.61	0.0072	107.26	—
4	0.549	3,086.67	0.0098	62.28	✓ Selected
5	0.552	3,660.02	0.0109	40.31	—

WCSS = within-cluster sum of squares (inertia); lower values indicate tighter clusters. k = 4: Hierarchical concordance = 100%; quartile concordance = 85.1%.

Supplementary Table S2. Stimulation Protocol Variables Across TSH Clusters

All stimulation protocol variables were balanced across the four TSH clusters. No significant differences were observed in gonadotropin dose, stimulation duration, peak estradiol, protocol type (agonist vs. antagonist), or gonadotropin type (all $p > 0.05$), confirming that stimulation protocol was not a confounding variable in the relationship between TSH cluster and clinical pregnancy outcome.

Variable	Group I (TSH 0–1) n=191	Group II (TSH 1–2) n=290	Group III (TSH 2–3) n=250	Group IV (TSH 3–4) n=147	p-value
Gonadotropin ampoules, median (IQR)	36 (28–45)	35 (26–49)	36 (26–49)	33 (24–47)	0.785 a
Stimulation duration (days),	11 (10–12)	11 (10–12)	11 (10–12)	10 (10–12)	0.447 a

Variable	Group I (TSH 0–1) n=191	Group II (TSH 1–2) n=290	Group III (TSH 2–3) n=250	Group IV (TSH 3–4) n=147	p-value
median (IQR)					
Peak E2 (pg/mL), median (IQR)	2198 (1674–2970)	2362 (1749–3000)	2560 (1658–3008)	2156 (1507–2964)	0.355 a
**Protocol type, n (%)**					
Short agonist	116 (60.7%)	176 (60.7%)	158 (63.2%)	84 (57.1%)	0.698 b
Short antagonist	75 (39.3%)	114 (39.3%)	92 (36.8%)	63 (42.9%)	
**Gonadotropin type, n (%)**					
Folisurge	75 (39.3%)	117 (40.3%)	91 (36.4%)	55 (37.4%)	0.923 b
GonalF	56 (29.3%)	74 (25.5%)	76 (30.4%)	39 (26.5%)	
Combined/other	60 (31.4%)	99 (34.1%)	83 (33.2%)	53 (36.1%)	

IQR = interquartile range; E2 = estradiol; TSH = thyroid-stimulating hormone. 1 ampoule = 75 IU. a Kruskal-Wallis test. b Chi-square test. All p-values > 0.05 (non-significant).

Supplementary Figures

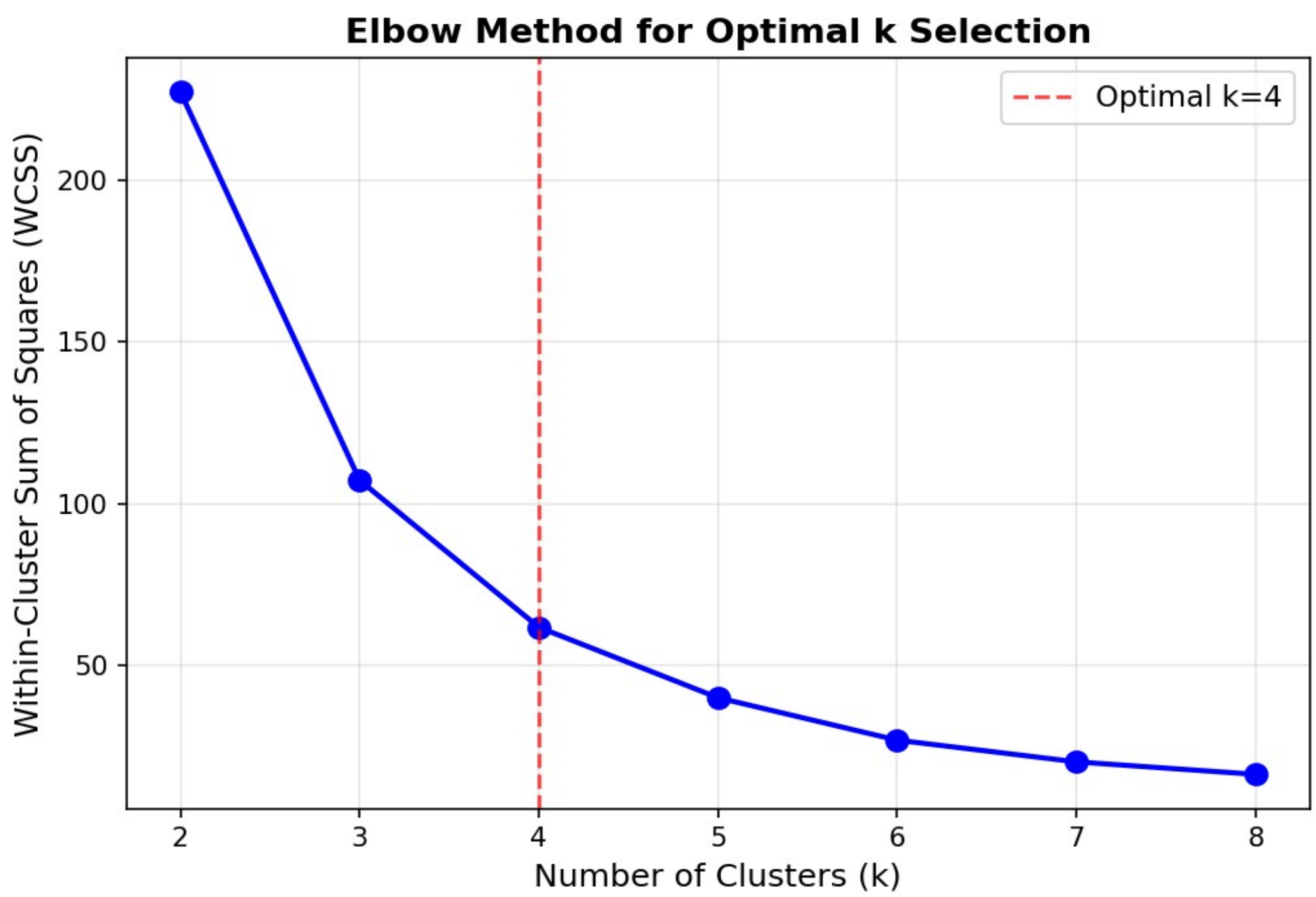

Supplementary Figure S1. Elbow plot showing within-cluster sum of squares (WCSS) as a function of the number of clusters (k = 1 to 10). The inflection point at k = 4 supports selection of four TSH clusters.

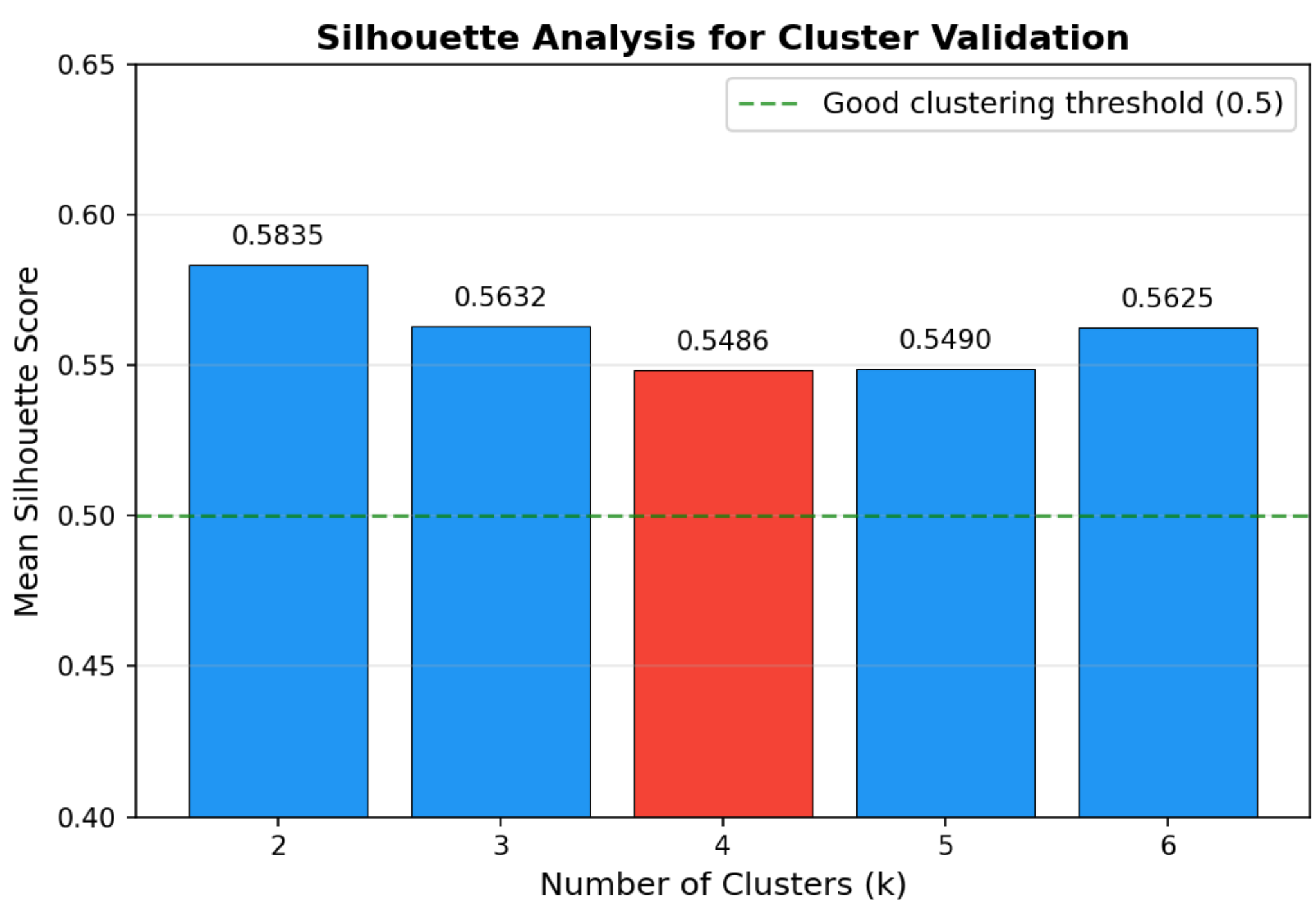

Supplementary Figure S2. Mean silhouette coefficient for k = 2 to 8 clusters. Values above 0.5 indicate reasonable cluster structure. k = 4 (silhouette = 0.549) was selected based on the combination of clinical interpretability and highest Calinski-Harabasz index.

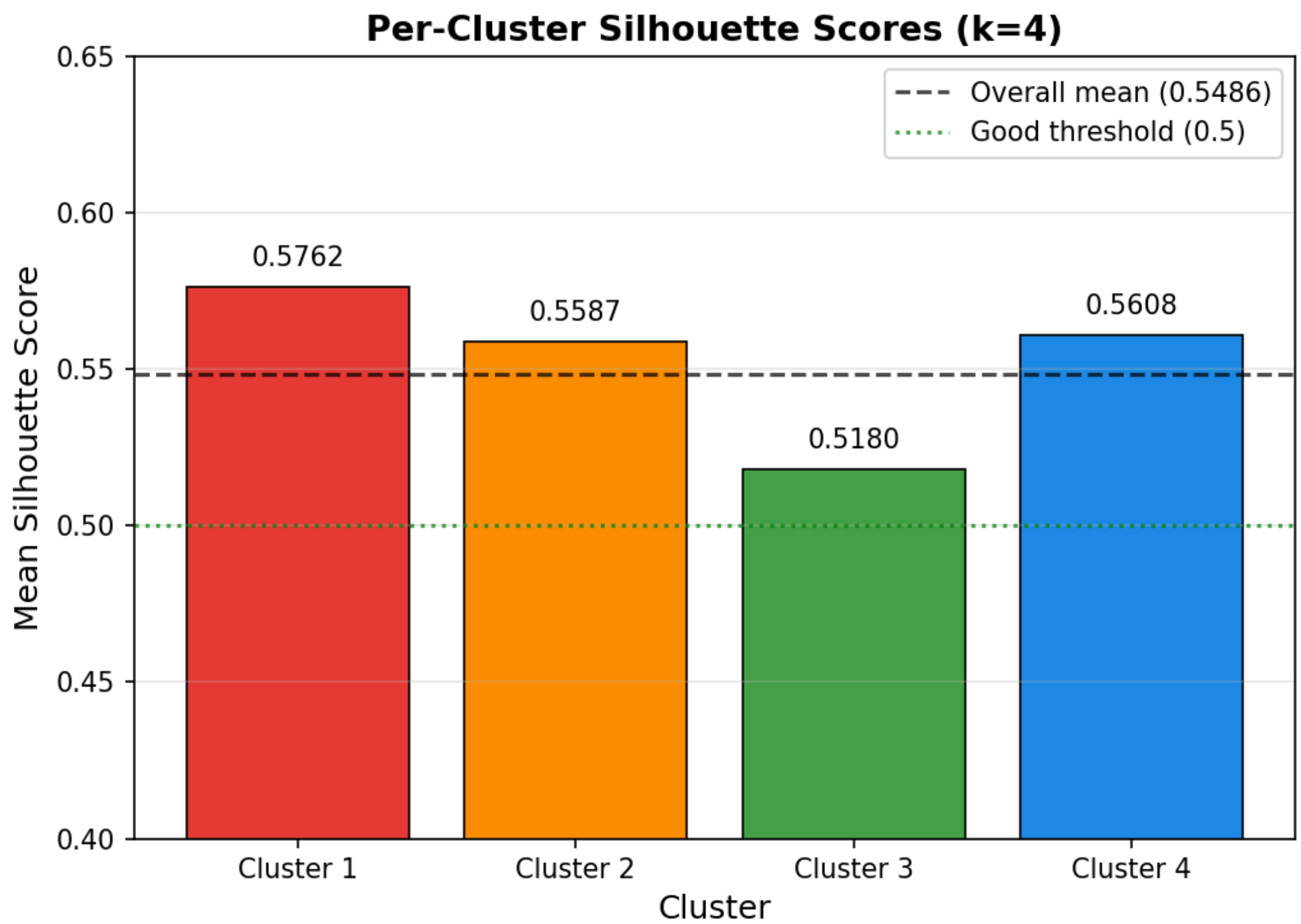

Supplementary Figure S3. Silhouette plot for k = 4 clusters showing per-sample silhouette coefficients. All four clusters have positive mean silhouette scores with minimal negative values, confirming adequate cluster separation.

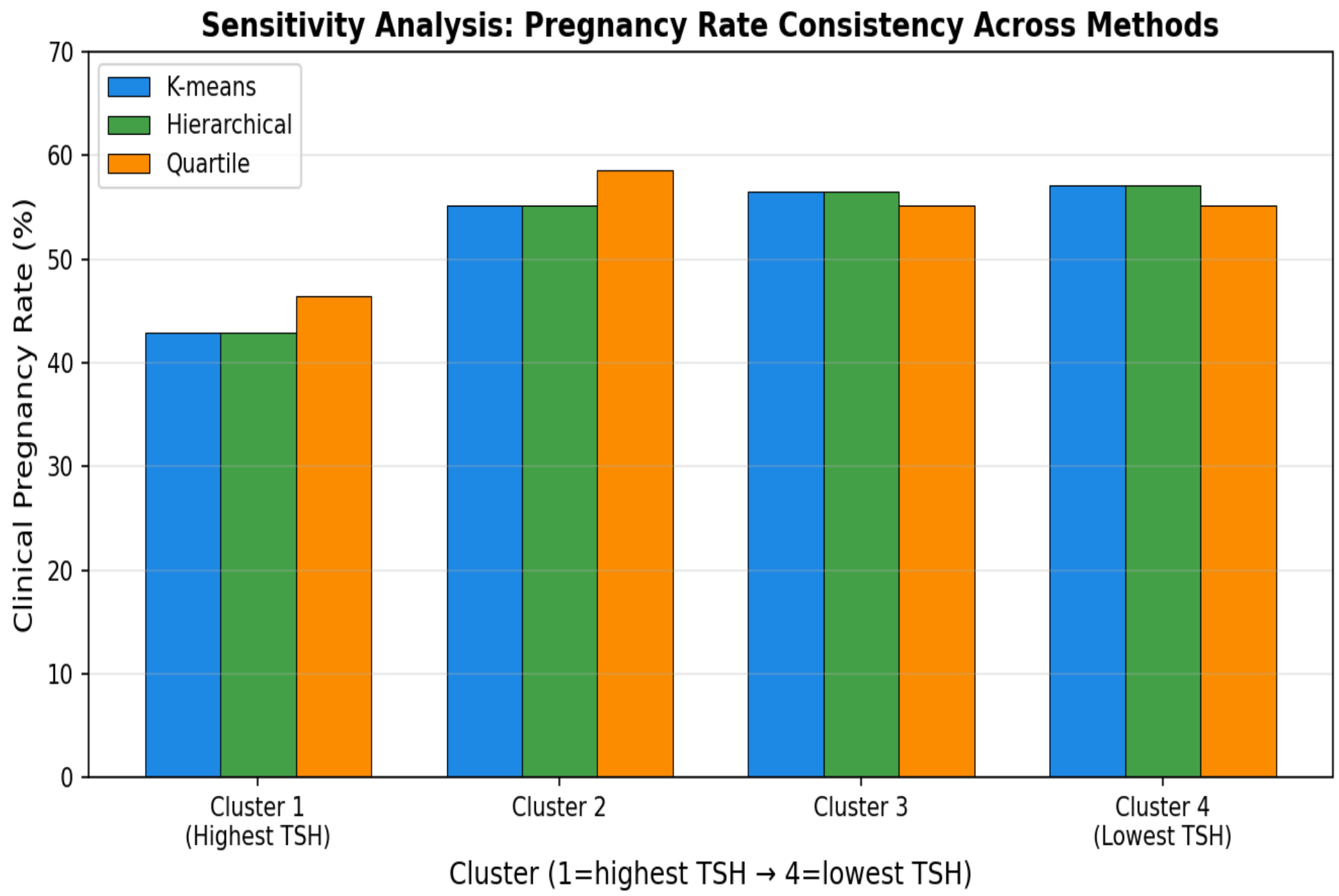

Supplementary Figure S4. Sensitivity analysis showing cluster pregnancy rates under alternative k values (k = 2, 3, 4, 5). The monotonic decrease in clinical pregnancy rate with increasing TSH is consistent across all cluster configurations, confirming the robustness of the main finding.

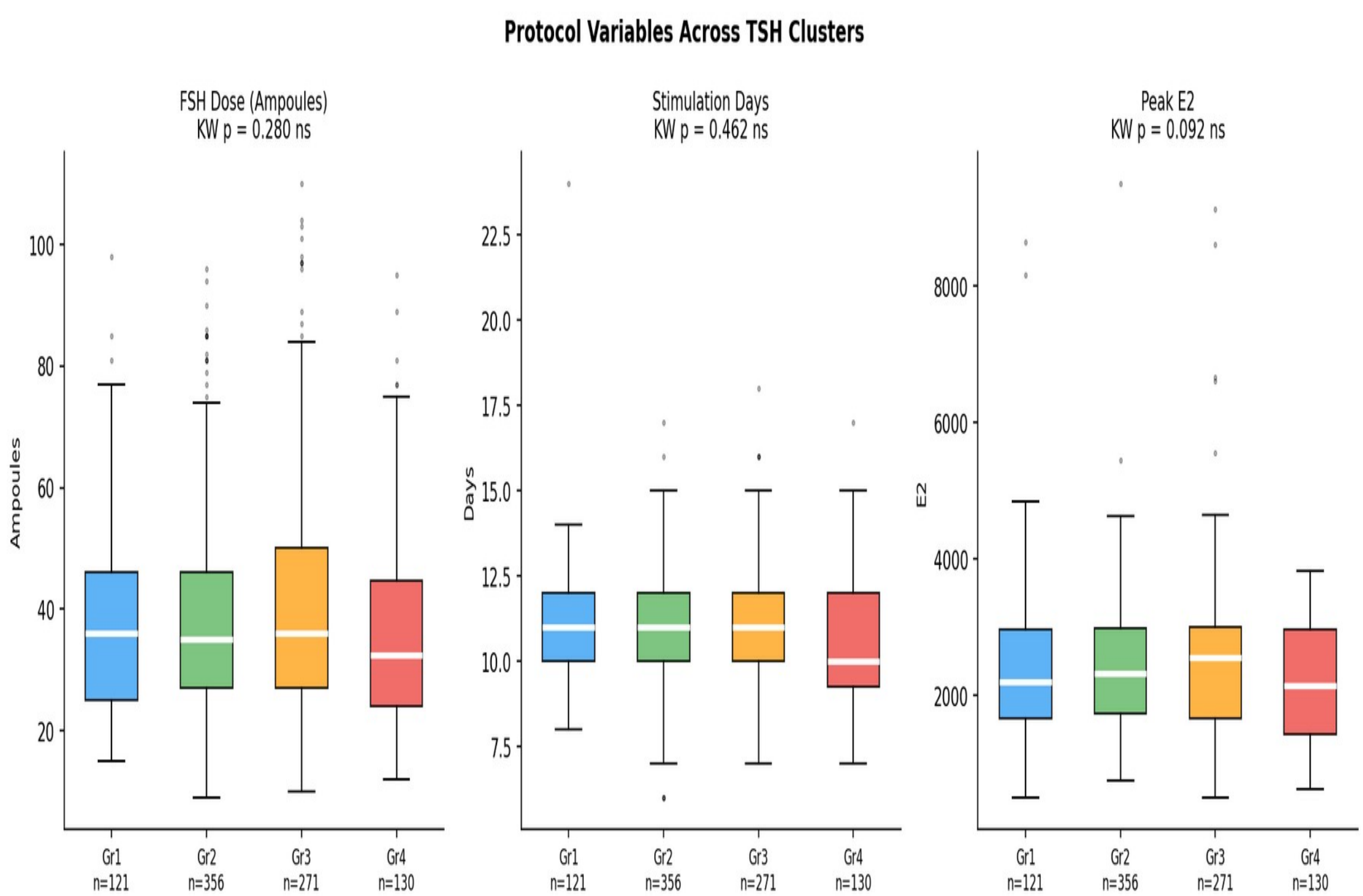

Supplementary Figure S5. Box plots of continuous stimulation protocol variables (gonadotropin ampoules, stimulation days, and peak estradiol) across the four TSH clusters. No significant differences were observed (Kruskal-Wallis, all p > 0.05), confirming that stimulation protocol did not confound the TSH-pregnancy association.

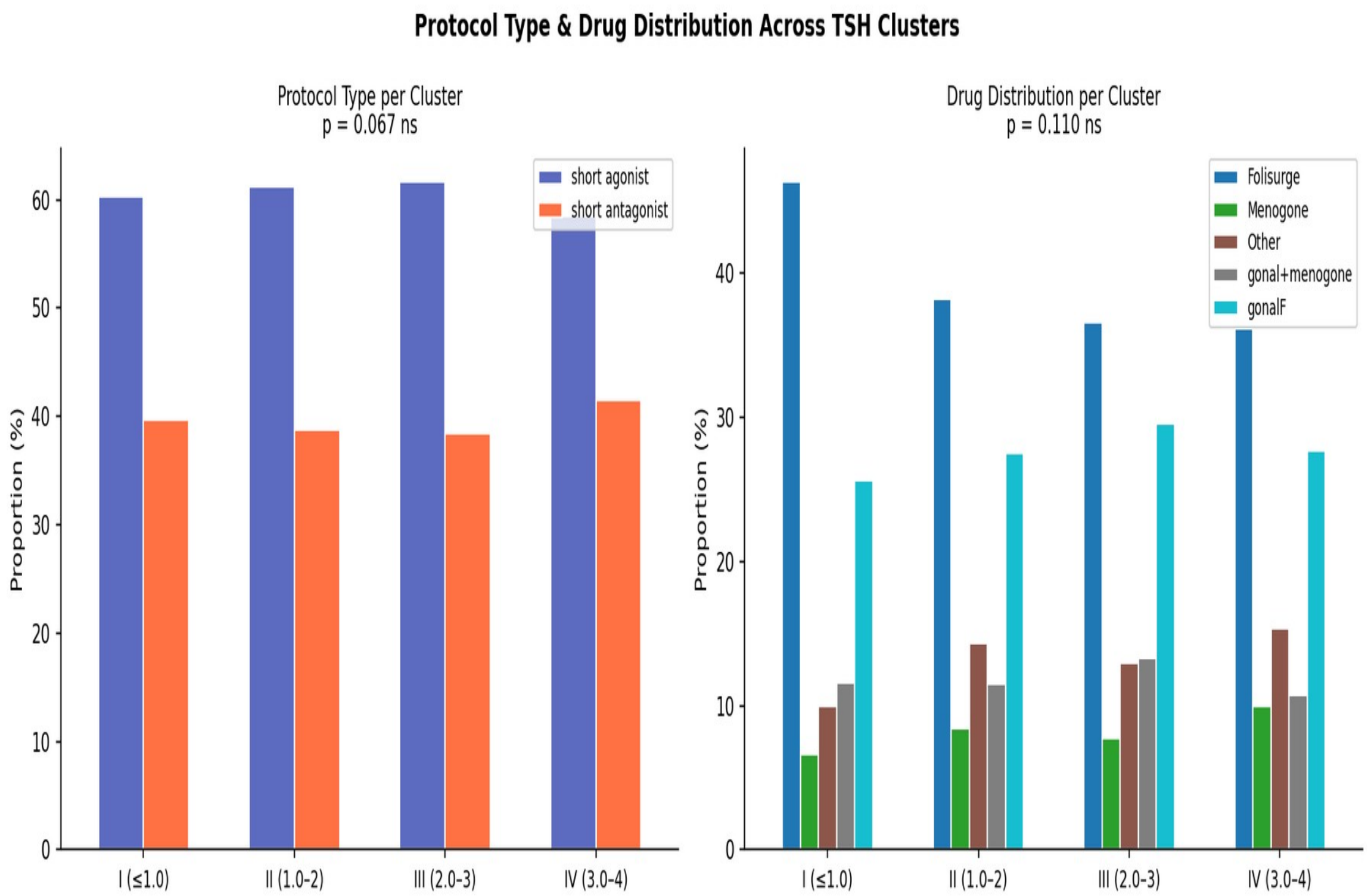

Supplementary Figure S6. Distribution of protocol type (short agonist vs. short antagonist) and gonadotropin agent across the four TSH clusters. The balanced distribution across clusters (Chi-square p > 0.05) confirms no systematic difference in treatment protocols between TSH groups.

All analyses were conducted in R (v4.2) and Python (v3.10). K-means clustering was performed using the 'stats' package in R and validated using the 'cluster' and 'clusterCrit' packages. Protocol statistics were computed using numpy and pandas.
